# Inflammasome complex genes with clinical relevance suggest potential as therapeutic targets for anti-tumor drugs in clear cell renal cell carcinoma

**DOI:** 10.1515/biol-2022-0980

**Published:** 2024-11-12

**Authors:** Fengchao Yin, Fang Li, Pan Qi, Aili Zhang

**Affiliations:** Department of Urology, The Fourth Hospital of Hebei Medical University, Shijiazhuang, Hebei, China; Department of Urology, Shijiazhuang People’s Hospital, Shijiazhuang, Hebei, China; Department of Pathology, The Fourth Hospital of Hebei Medical University, Shijiazhuang, Hebei, China

**Keywords:** clear cell renal cell carcinoma, inflammasome complexes, prognosis, immune microenvironment, cancer therapy

## Abstract

Clear cell renal cell carcinoma (ccRCC) is a challenging malignancy characterized by intricate biology and clinical characteristics. Despite advancements in treatment strategies, the molecular mechanisms underlying ccRCC initiation, progression, and therapeutic resistance remain elusive. Inflammasomes, multi-protein complexes involved in innate immunity and inflammation, have emerged as potential regulators in cancers. However, their involvement and mechanisms in ccRCC remain poorly understood. In this study, we conducted a systematic investigation into the expression patterns and clinical significance of inflammasome complexes in ccRCC. We found the perturbation of inflammasome complexes genes was related to patient’s prognosis and other clinical characteristics. By developing an Inflammasome Complexes (IFC) score and identifying IFC subtypes with distinct clinical characteristics and oncogenic roles, our study suggested that inflammasome activation could impact tumorigenesis and modulate the tumor immune landscape, particularly its positive correlations with immunosuppressive macrophages. Furthermore, our study revealed the potential of inflammasome complex genes as predictive markers for patient responses to various anti-tumor drugs, including Osimertinib, Ulixertinib, Telomerase Inhibitor IX, and GSK2578215A. These findings have significant clinical implications and offer opportunities for guiding treatment strategies and improving patient outcomes of ccRCC.

## 1 Introduction

Clear cell renal cell carcinoma (ccRCC) is one of the most prevalent malignant renal tumors, posing a significant challenge due to its intricate biology and clinical characteristics [1,2]. Given its complex nature and global clinical impact, ccRCC has become a critical focal point for researchers and clinicians [3]. The ccRCC exhibits distinct histological traits that underlie its propensity for high metastatic potential, aggressiveness, and inherent resistance to conventional therapeutic interventions [4,5]. Despite the advancement of clinical treatment strategies, a comprehensive understanding of the precise molecular mechanisms governing ccRCC initiation, progression, and the development of therapeutic resistance remains elusive.

The inflammasome, a multifaceted multi-protein complex [6], stands as a key player in the coordination of inflammation and innate immunity, serving as a vital component in host defense against pathogenic microorganisms. Upon activation, these complexes drive the maturation and subsequent secretion of pro-inflammatory cytokines [7,8]. This activation process not only facilitates a robust immune response but also contributes to the resolution of infections [9]. Intriguingly, inflammasome complexes extend beyond immune cells and are detected in various non-immune cell types, including cancer cells [10,11]. Recent years have unveiled compelling evidence showcasing the connection between inflammasome signaling dysregulation and the genesis of multiple malignant tumors [12,13]. However, the involvement and mechanism of the inflammasome in clear cell renal cell carcinoma remains unclear.

Tumor immune microenvironment (TIME) has gained popularity recently as a critical factor influencing cancer progression and treatment response [14]. In this complex environment, inflammatory complexes exert various significant effects on the TIME [15,16]. The activation of these inflammasome complexes within tumor cells stimulates the release of pro-inflammatory cytokines and manipulates the modulation of the immune landscape, thus having a widespread impact on several aspects of tumorigenesis [17,18]. The efficacy of immunotherapy has achieved remarkable success across certain cancers [19,20]. Inflammasome complex participation is a particularly interesting topic in the realm of cancer therapies [21]. Moreover, research efforts have focused on the interplay between inflammatory signaling and the response to chemotherapy and target therapy [16]. Certain anti-tumor drugs have demonstrated the ability to incite inflammasome activation within tumor cells [22]. This activation of the inflammasome might subsequently increase tumor susceptibility to specific drugs. Elucidating the mechanisms by which the inflammasome complex regulates the interaction between the tumor and immune microenvironment, as well as any possible implications for therapeutic responses, is critical to the development of effective treatments for ccRCC.

In this study, we provided a comprehensive analysis of the expression patterns and clinical implications of inflammasome complexes in ccRCC. By generating an Inflammasome Complex (IFC) score and identifying IFC subtypes, we gained insights into the immune microenvironment of ccRCC, particularly its associations with immunosuppressive macrophages. Furthermore, our findings revealed the potential of IFC genes as predictive markers for patients’ responses to immunotherapy, chemotherapy, and target-therapy. These results helped to clarify the role of inflammasome complexes in ccRCC, and have the potential to direct therapeutic approaches and enhance patient outcomes of ccRCC.

## 2 Materials and methods

### 2.1 Data collection and integration

A total of 15 IFC genes were collected from a previous study [23], including NLRP1, NLRP3, CASP4, CASP5, NLRC5, NLRP6, NLRP12, NLRP7, NAIP, NLRC4, AIM2, IFI16, MEFV, NLRP2, and PYCARD. FPKM-UQ normalized RNA-Seq expression profile of The Cancer Genome Atlas (TCGA) kidney renal clear cell carcinoma patients was downloaded from UCSC Xena (https://xenabrowser.net/) platform [24], including 535 tumorous samples and 72 adjacent non-tumorous tissues. The expression value of genes was log-transformed for further analysis. Clinical information of patients, including age, gender, overall survival (OS), progression-free interval (PFI), histological grade, and pathological stages, was also obtained from the Xena platform.

### 2.2 Immunohistochemistry

In order to evaluate the expression of inflammatory complex in ccRCC, we selected NLRP family members, *NLRP1*, *NLRP3*, and *NLRP*, for experiments, and detected the expression of these three proteins in ccRCC and adjacent tissues. A total of 30 patients with ccRCC were collected from the urological department of the Fourth Hospital of Hebei Medical University from January 2016 to June 2017. Meanwhile, adjacent tissues were routinely retained 2 cm away from the tumor margin. All tissues were routinely embedded in paraffin wax and confirmed by pathology. Five white slices of each paraffin-embedded tissue block were cut continuously with a thickness of 4 μm, and immunohistochemical staining was performed. *NLRP1* and *NLRP3* (Abcam, Cambridge, UK), both in 1:100 dilution. *NLRP6* (Millipore), diluted in 1:1,000. Each group of slices was incubated with diluted *NLRP1*/*NLRP3*/*NLRP6* at 4°C overnight. Sections were coupled with horseradish peroxidase-coupled antibodies (Abcam) at 1:500 dilution at room temperature for 2 h. Then 3,3-diaminobenzidine is added. The staining was observed under optical microscope (ZEISS). Immunohistochemical scores were determined by two pathologists with diagnostic experience. The scoring method was the combination of staining intensity and proportion of positive staining area. The scoring criteria of staining intensity were negative score 0; weak positive marks 1 point; medium positive score 2 points; strong positive score 3 points. The number of stained cells was scored according to the proportion of positive cells in each section, and the percentage of positively stained cells in the total number of cells was taken as the average number of each field score: the score of no positive staining was 0; positive staining of 0–20% was l; positive staining of 20–75% was 2; positive staining greater than 75% was 3. Total score = positive percentage score × staining intensity score. A score of 0–4 was low expression, and a score of 5–9 was high expression.


**Informed consent:** Informed consent has been obtained from all individuals included in this study.
**Ethical approval:** The research related to human use has been complied with all the relevant national regulations, institutional policies and in accordance with the tenets of the Helsinki Declaration, and has been approved by the authors’ institutional review board or equivalent committee.

### 2.3 Mutation analysis

The mutation annotation format files of somatic mutation data of ccRCC patients were obtained from the TCGA MC3 project [25]. We used the R package “maftools” to calculate the tumor mutation burden (TMB) for ccRCC samples [26].

### 2.4 Survival analysis

We used R package “survival” and “survminer” to apply uni- and multi-variate Cox regression models based on the expression level of IFC genes. Kaplan–Meier (KM) curves were used to compare the survival differences between distinct patient groups.

### 2.5 Calculation of the IFC score and identification of IFC groups

Single-sample GSEA (ssGSEA) algorithm was applied using R package “GSVA” [27] to calculate the IFC score in each ccRCC sample based on the expression level of 15 IFC genes. Next, all samples were divided into two groups (high and low) based on the median value of IFC scores.

### 2.6 Differential expression analysis of protein-coding genes

The annotation of protein-coding genes was obtained from GENCODE v22 [28] (https://www.gencodegenes.org/). We used the R package “limma” to identify dysregulated genes between distinct sample groups [29]. Genes with empty expression values in more than 50% of samples were filtered out for further analysis. Genes with Benjamini and Hochberg (BH) generated false discovery rate (FDR) values less than 0.01 and were identified as differentially expressed.

### 2.7 Functional enrichment analysis

Ten oncogenic pathways and ten cancer hallmark gene sets were collected from previous studies [30,31]. We downloaded the hallmark gene sets representing various biological processes from MSigDB [32] (https://www.gsea-msigdb.org/gsea/msigdb). The ssGSEA algorithm was applied to calculate the enrichment level of each oncogenic pathway and hallmark gene set across ccRCC samples. Metascape platform (https://metascape.org/) was used to perform Gene ontology (GO), Kyoto Encyclopedia of Genes and Genomes (KEGG) pathway, and Reactome gene set enrichment analysis [33].

### 2.8 Identification of the IFC-based subtypes of ccRCC patients

Using the expression of 15 IFC genes, we performed unsupervised non-negative matrix factorization (NMF) [34] clustering on ccRCC patients. R package “NMF” was used to stratify ccRCC samples into two subtypes according to the cophenetic correlation coefficient [35]. Other parameters were set as follows: method = “brunet”, cluster number *k* = 2:10, nrun = 50.

### 2.9 The tumor immune microenvironment patterns of ccRCC

We applied Estimation of Stromal and Immune cells in Malignant Tumor tissues using the Expression data (ESTIMATE) method using the R package “estimate” to evaluate the immune cell abundance, stromal cell abundance, tumor purity, and overall tumor immune microenvironment of ccRCC samples [36]. A previous study defined 29 gene signatures representing tumor microenvironment. We collected these signatures and calculated the enrichment level of these signatures using the ssGSEA algorithm. Unsupervised clustering was performed on ccRCC by TIME signatures to define distinct immune clusters. Immune cell infiltration estimation of TCGA ccRCC samples generated by various methods [37,38,39] was downloaded from TIMER 2.0 [40] (http://timer.cistrome.org/). The marker genes of various immune cell types were collected from CellMarker 2.0 [41] (http://bio-bigdata.hrbmu.edu.cn/CellMarker).

### 2.10 Prediction of immunotherapy, chemotherapy, and target-therapy responses

Tumor Immune Dysfunction and Exclusion (TIDE, http://tide.dfci.harvard.edu/) defined “Dysfunction scores” and “Exclusion scores” representing the dysfunction of T cell and prevention of T cell infiltration, respectively [42]. These two scores could evaluate primary mechanisms of tumor immune evasion, thus representing the possibility of immunotherapy response. The gene expression profile of ccRCC cell lines and drug sensitivity data of chemotherapy were downloaded from the Genomics of Drug Sensitivity in Cancer [43] (https://www.cancerrxgene.org/) database. Based on the median value of ln(IC_50_) value of each drug, we divided ccRCC cells into two groups (sensitive and resistant), and then we identified dysregulated IFC genes between two groups and considered them as potential drug targets.

### 2.11 Statistical analysis

The statistical tests employed to analyze data in this study include the Mann–Whitney *U* test was utilized to compare the non-normally distributed variables between two groups. The Kruskal–Wallis test was employed to assess the association between the IFC score and different pathological stages of ccRCC. Pearson’s correlation coefficient and Spearman’s correlation coefficient were computed to explore the potential relationships between two variables. Fisher’s exact test was employed to perform overlap analysis. All statistical analyses were performed using R statistical software.

## 3 Results

### 3.1 Dysregulated IFC genes were correlated with different clinical outcomes in ccRCC

**Figure 1:**
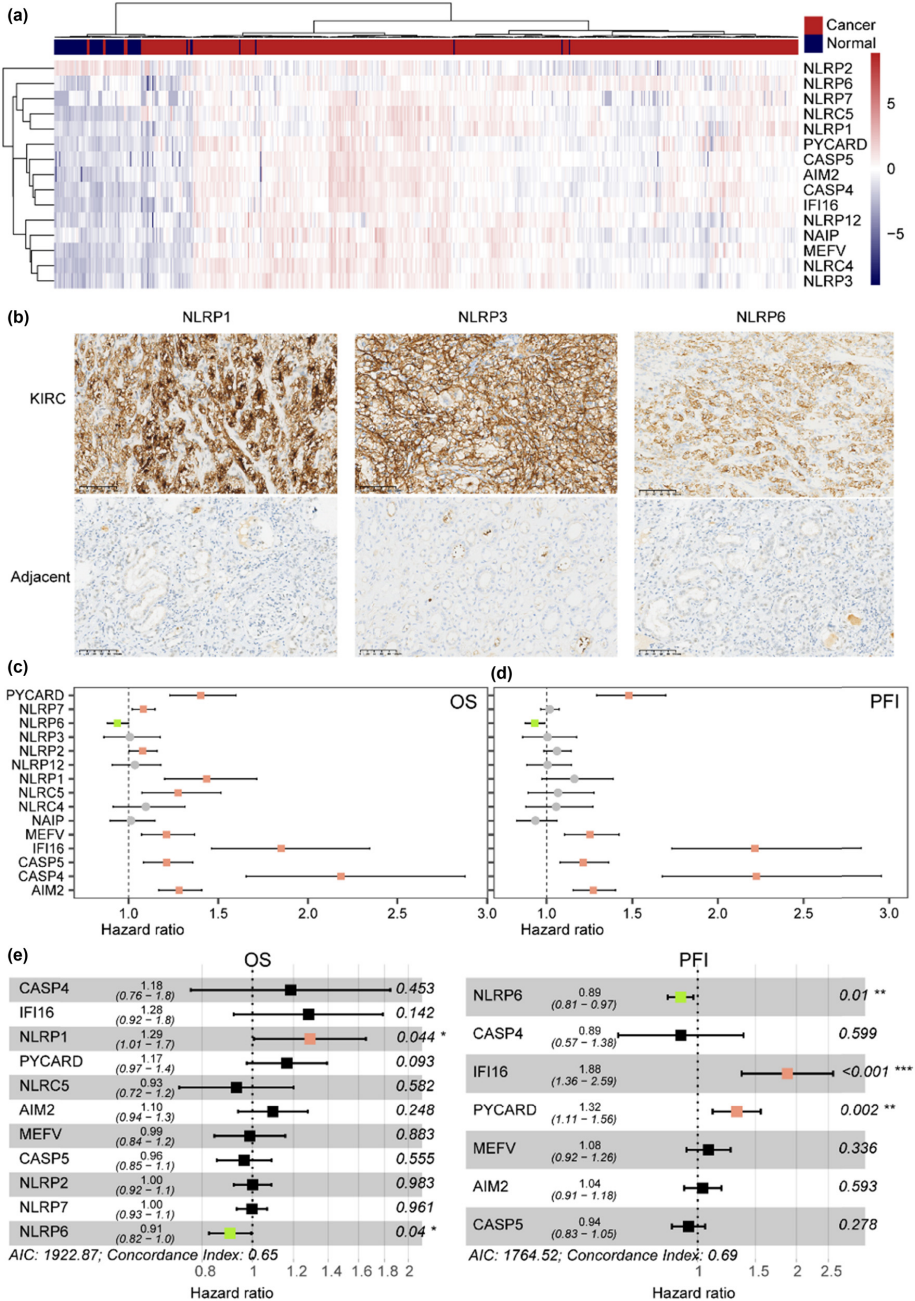
Expression pattern and survival relevance of inflammasome complex (IFC) genes. (a) Heatmap illustrates the expression levels of IFC genes in ccRCC samples compared to normal samples. The color scale represents gene expression levels, with red indicating upregulation and blue indicating downregulation. (b) Expression of NLRP1, NLRP3, and NLRP6 in ccRCC and adjacent tissues (IHC 200 times magnification). (c and d) Forest plots of uni-variate Cox regression model for OS and PFI in ccRCC patients. The plot displays hazard ratios and corresponding 95% confidence intervals for each IFC gene. Red bars are associated with worse survival outcomes, while green bars are associated with better survival outcomes. (e) Forest plots of multi-variate Cox regression model.

All of the IFC genes were found to be differentially expressed in tumor samples (Figure 1a). All IFC genes, except for *NLRP2*, showed significant elevated levels in tumor samples than in normal samples (Mann–Whitney *U* test *p* < 0.05) (Figure S1a). A clear separation was found between ccRCC and normal samples based on the expression levels of IFC genes, suggesting a potential perturbated expression pattern during tumorigenesis. The results from IHC showed that the expression rate of *NLRP1*, *NLRP3*, and *NLRP6* in ccRCC tissues was significantly higher than that in adjacent non-tumorous tissues (Figure 1b, Table S1). Next, survival analysis revealed significant correlations between IFC gene expression and ccRCC clinical outcomes. Higher level of *NLRP6* expression was associated with a prolonged OS. However, higher level of *PYCARD*, *NLRP7*, *NLRP2*, *NLRP1*, *NLRP5*, *MEFV*, *IFI16*, *CASP5*, *CASP4*, and *AIM2* might lead to worse OS (Figure 1c). Additionally, some of these IFC genes were also found to have an impact on patients’ PFI (Figure 1d). In order to identify independent prognostic factors, we also performed a multi-Cox regression analysis. Results showed that *NLRP6* could serve as a protective factor for both OS and PFI, whereas *NLRP1*, *IFI16*, and *PYCARD* served as risky factors for OS and PFI, respectively (Figure 1e). In summary, our results revealed significant dysregulation of inflammasome complexes genes, suggesting their potential roles in ccRCC development and patient prognosis.

### 3.2 Significant correlations were revealed between IFC score and clinical features

**Figure 2:**
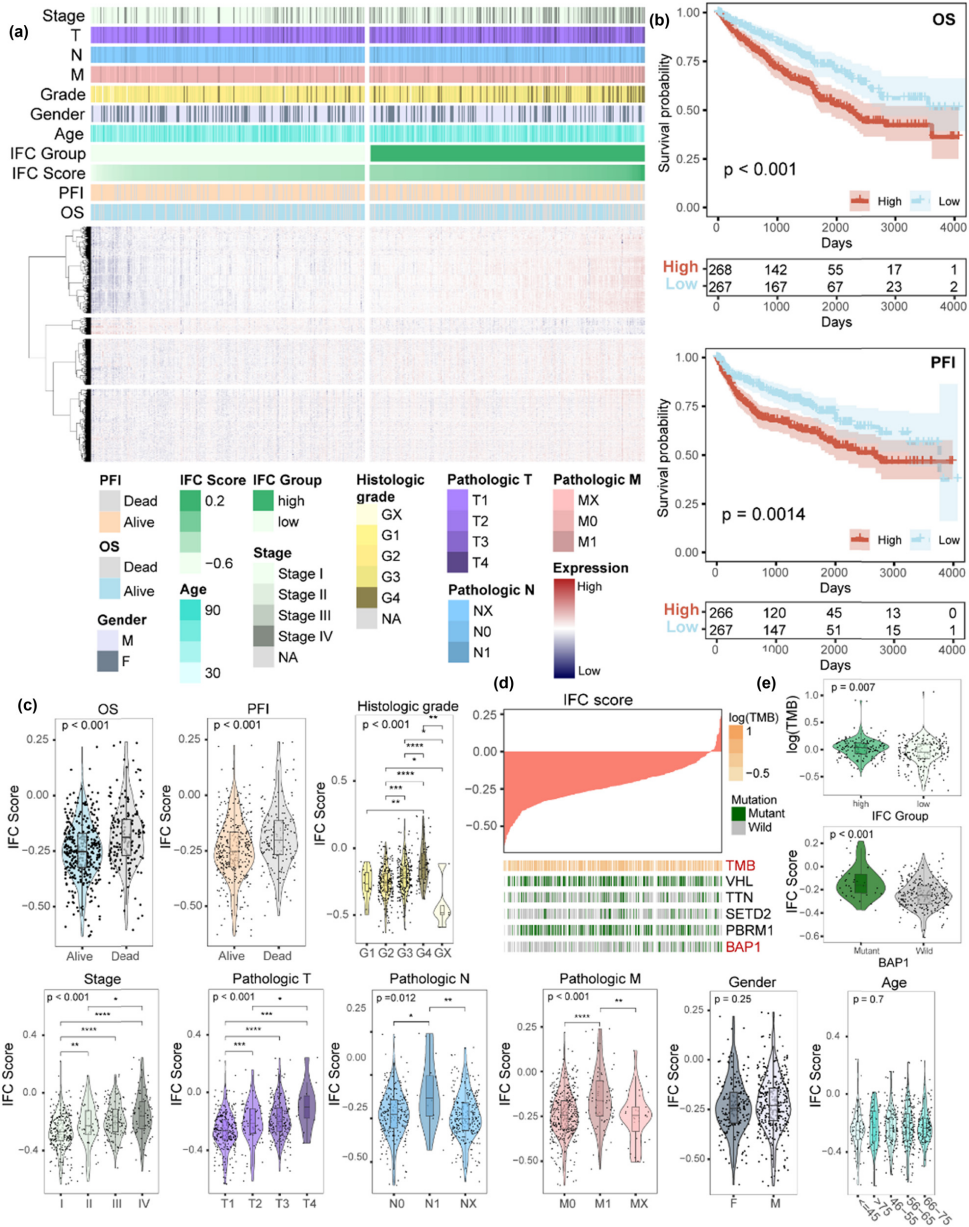
The clinical features associated with the IFC score in ccRCC patients. (a) Heatmap illustrates the expression patterns of dysregulated protein-coding genes between the high and low IFC groups, along with the corresponding clinical characteristics of the samples. (b) KM survival curves demonstrate the differences in OS and PFI between the high and low IFC groups. (c) Violin plots show the distribution of IFC scores among different clinical groups, including gender, age, survival status, pathological stages, and histologic grades (Mann–Whitney *U* test *p* < 0.05; Kruskal–Wallis test *p* < 0.05). (d) An overview of the association between IFC score and mutation status of the genes with high-frequency somatic mutation and TMB. (e) Violin plots show the TMB differences between high and low IFC groups and the distribution of IFC scores among BAP1 mutant and wild-type samples.

Here, we calculated the IFC score across ccRCC cancer samples using the ssGSEA algorithm based on the expression level of 15 IFC genes. The samples were ranked and divided into high and low IFC groups according to the median value of IFC scores (Figure 2a). KM curves showed that patients from the low IFC group had a prolonged OS and PFI than patients from the high IFC group (Figure 2b). We further explored the clinical associations of distinct IFC groups. Results showed a significant increase in the IFC score as the pathologic stages and grades advanced (Figure 2c). However, no significant differences in the IFC score were observed across different genders and age groups. In addition, we collected genes with a high-frequency somatic mutation in ccRCC and calculated the TMB of the samples (Figure 2d, Figure S1b and c). Subsequently, we investigated the association between the IFC score and gene mutations. We observed an elevated tumor mutational burden in the high IFC group. Moreover, the IFC score was higher in *BAP1*-mutant samples than in *BAP1*-wild-type samples (Figure 2e). Previous research has concentrated on the significance of *BAP1* mutation status in ccRCC [44,45,46]; our results suggest that inflammasome complexes genes might exert an impact on the interplay between *BAP1* and ccRCC development. In summary, our findings revealed a significant correlation between IFC scores, increased TMB, and certain clinical characteristics in ccRCC.

### 3.3 Dysregulated genes between high and low IFC groups were involved in distinct biological functions

**Figure 3:**
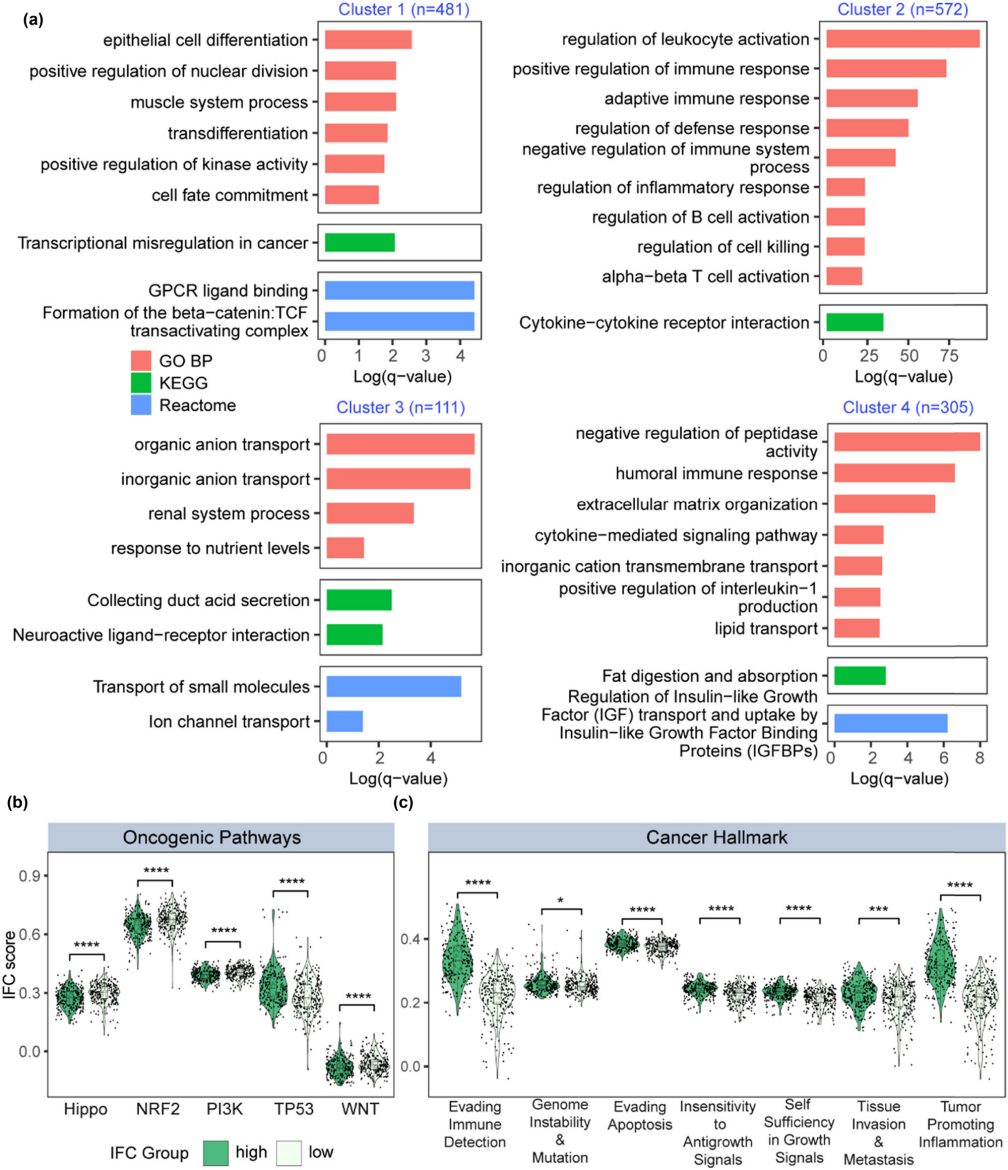
Functional enrichment analysis of high and low IFC groups. (a) Barplot illustrates the results of enrichment analysis for GO, KEGG, and Reactome gene sets. (b) Violin plots demonstrate the differences in oncogenic pathway activation levels between the high and low IFC groups (Mann–Whitney *U* test *p* < 0.05). (c) Violin plots depict the differential expression of cancer hallmark gene sets between the high and low IFC groups (Mann–Whitney *U* test *p* < 0.05; Kruskal–Wallis test *p* < 0.05).

We performed differential expression analysis and identified dysregulated protein-coding genes (110 down-regulated genes and 1361 up-regulated genes, fold change > 2 and FDR < 0.01) between high and low IFC groups (Figure 2a). Dysregulated genes were divided into four clusters by an unsupervised hierarchical clustering method. Then, we performed functional enrichment analysis on the four gene clusters, respectively (Figure 3a). Cluster 1 genes were enriched in tumor invasion, metastasis, and proliferation-related process, including epithelial cell differentiation, positive regulation of nuclear division, and GPCR ligand binding. Cluster 2 genes were involved in biological processes regarding tumor immune response, immune regulation, and formation of tumor microenvironment. Cluster 3 genes were related to abnormal renal function and metabolic regulation. Cluster 4 genes were enriched in terms that were related to various aspects, including renal dysfunction, metabolic regulation, tumor biological behavior, and immune regulation. The results from functional enrichment analysis implied the potential relationship among inflammasome signaling, immune landscape, renal development and tumorigenesis of ccRCC. In addition, we collected ten oncogenic pathways and ten cancer hallmark gene sets and quantified the enrichment level of these gene sets. As a result, we found that Hippo, NRF2, PI3K, TP53, and WNT pathways showed a dysregulated pattern between high and low IFC groups (Figure 3b). Seven cancer hallmark gene sets were significantly up-regulated in the high IFC group (Figure 3c), further suggesting the oncogenic roles of inflammasome signaling. In summary, our finding suggested a potential involvement of the inflammasome complex genes in the pathogenesis and progression of ccRCC.

### 3.4 Patients’ stratification revealed correlations with distinct clinical features and immunotherapy response

**Figure 4:**
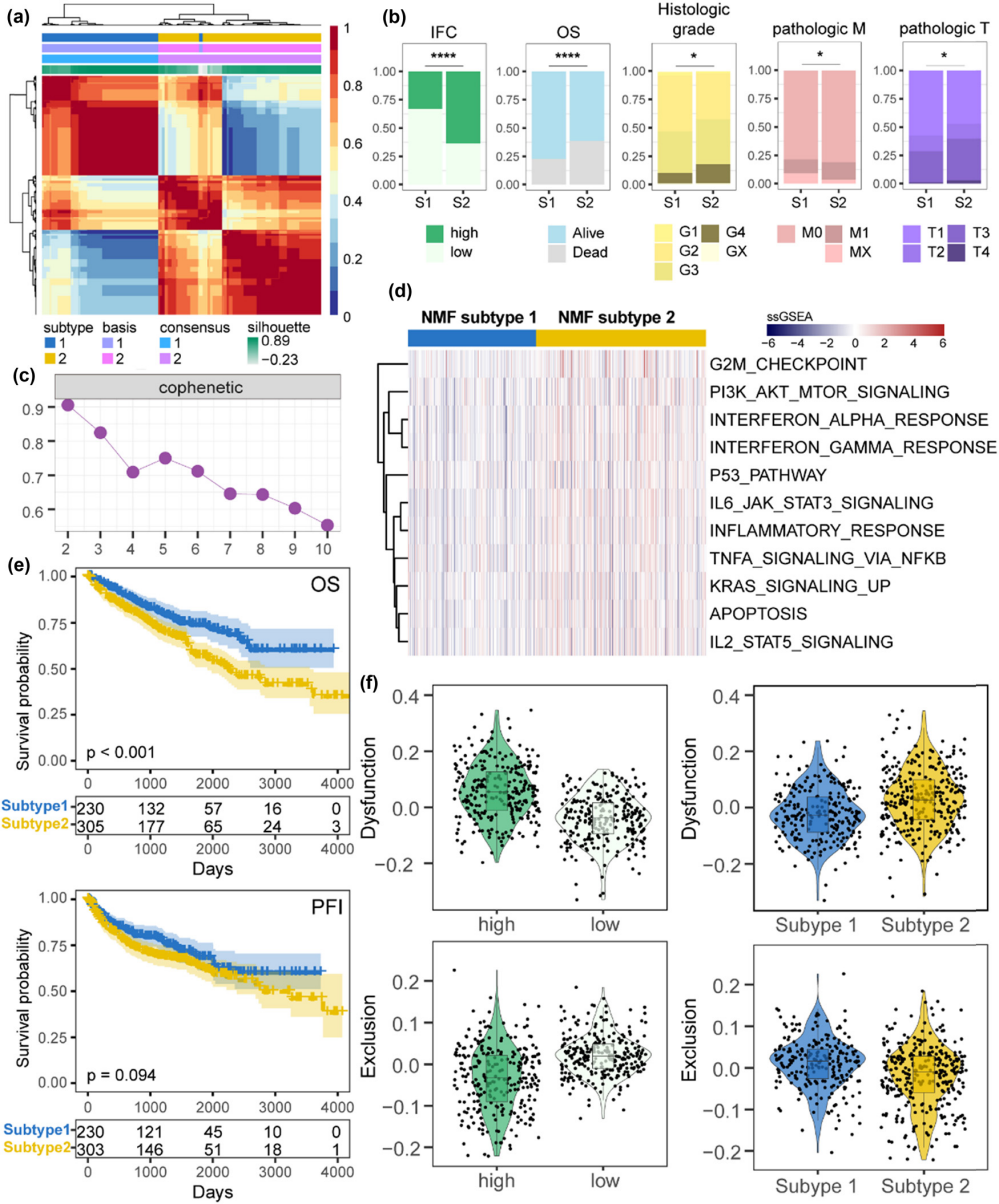
Identification and characterization of NMF-defined IFC subtypes. (a) Heatmap displays the results of NMF clustering (*k* = 2), which defined distinct IFC subtypes based on gene expression patterns. (b) The barplot illustrates the differences in clinical characteristics between the two IFC subtypes (Fisher’s exact test *p* < 0.05). (c) Cophenetic plot visualizing the quality of the NMF clustering results. (d) Heatmap shows the expression perturbations of functional gene sets between the two IFC subtypes. (e) KM curves demonstrate the differences in OS and PFI between the two IFC subtypes. (f) Violin plots display the differences in Exclusion score and Dysfunction score between the high and low IFC groups, as well as between the two IFC subtypes (Mann–Whitney *U* test *p* < 0.05).

Here, we sought to stratify ccRCC samples into clinically relevant molecular subtypes based on IFC genes. Applying NMF clustering, two IFC subtypes were identified through iterative analysis, including subtype 1 (*n* = 230) and subtype 2 (*n* = 305) (Figure 4a and c, Figure S1d). Patients from subtype 1 had better OS and PFI than patients from subtype 2 (Figure 4e). Moreover, we found that subtype 1 patients had relatively lower IFC scores than subtype 2 patients (Figure 4b). Then, we performed an overlap analysis to explore the clinical associations of distinct IFC subtypes. Results showed that patients with advanced pathologic stages and histologic grade were significantly enriched in subtype 2 (Figure 4b), suggesting a potential association between subtype 2 and more aggressive disease characteristics. To further characterize the subtypes, we performed differential expression analysis and identified 839 dysregulated genes, including 10 up-regulated and 829 down-regulated genes (fold change > 2 and FDR < 0.01) (Figure S1e). Next, we quantified various functional gene sets and compared the expression levels between two subtypes (Figure 4d). As a result, oncogenic and immune pathways showed significant up-regulation in subtype 2. Considering the strong associations between IFC subtypes, high/low IFC groups, and tumor immunity, we hypothesized that different patients may exhibit varying responses to immunotherapy. Thus, we calculated the Dysfunction score and Exclusion score of ccRCC samples using TIDE. Results showed that patients from subtype 1 and patients with lower IFC scores exhibited lower Dysfunction levels of score and higher levels of Exclusion scores, suggesting a worse chance to respond to immunotherapy (Figure 4f). In summary, our results demonstrate a strong correlation between IFC-based subtypes and the tumor immune characteristics.

### 3.5 IFC scores were positively correlated with immunosuppressive macrophages

**Figure 5:**
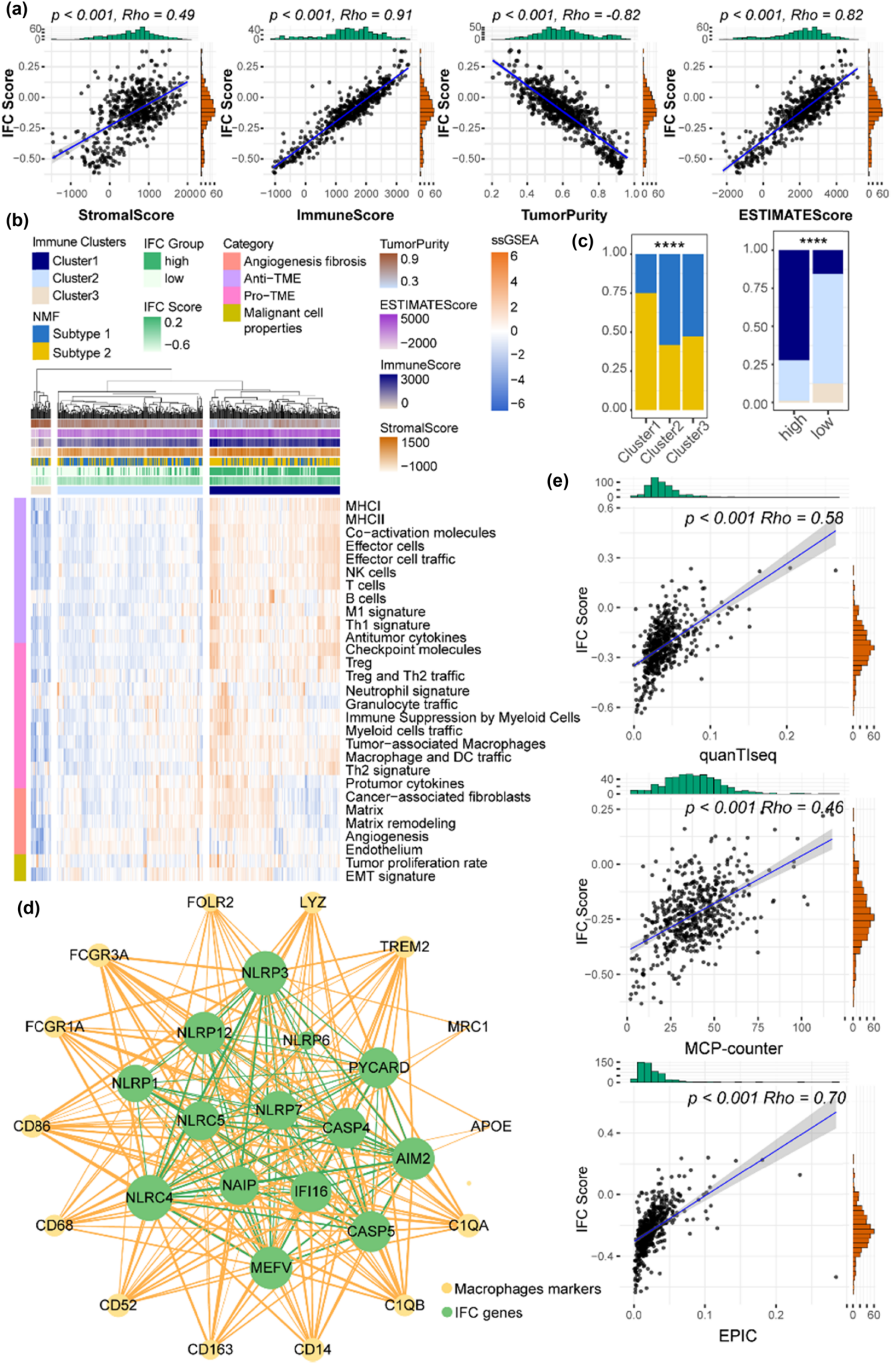
IFC scores were positively correlated with macrophage abundance. (a) Correlation curves display the correlation between the ESTIMATE score, immune score, stromal score, tumor purity, and IFC score. (b) TIME signatures defined three immune clusters. The heatmap displays the expression levels of 29 signatures across ccRCC samples. (c) Barplots illustrate the overlap among the three immune clusters, the high and low IFC groups, and the two IFC subtypes (Fisher’s exact test *p* < 0.05). (d) Co-expression network between IFC genes and macrophage marker genes. The network visualizes the co-expression relationships within IFC genes (shown in green edges), as well as between IFC and macrophage marker genes (shown in yellow edges). (e) Correlation curves show the correlation between the IFC score and the abundance of macrophages derived from three different data sources.

Here, we sought to further explore the relationship between IFC and TIME patterns. First, we evaluated ccRCC TIME using the ESTIMATE algorithm, and results showed that IFC scores were positively correlated with ESTIMATE, immune, and stromal scores, whereas they negatively correlated with tumor purity, suggesting increased infiltration levels of immune cells in high IFC score samples (Figure 5a). Next, we collected 29 immune signatures from a previous study and quantified their expression levels across ccRCC samples. ccRCC samples were divided into three immune clusters (high-immunity Cluster 1 *n* = 236, medium-immunity Cluster 2 *n* = 263, low-immunity Cluster 3 *n* = 36) by unsupervised hierarchical clustering method (Figure 5b). As shown in Figure 5c, Cluster 1 samples were enriched in NMF subtype 2 and high IFC Score group, whereas Cluster 3 samples showed a significant overlap with NMF subtype 1 and low IFC score group, suggesting a distinct immune landscape. In addition, we explored the relationship between IFC scores and various immune cell abundance. The strongest correlation was found between IFC scores and macrophage abundances (Figure 5e), suggesting a potential immunosuppressive microenvironment in ccRCC patients with high IFC scores. Thus, we constructed a co-expression network and found intense interplay between macrophage marker genes and IFC genes (Figure 5d), suggesting a potential functional relationship and shared regulatory mechanisms between macrophages and the inflammasome signaling in ccRCC. The relationship between changes in macrophage abundance and the immunotherapy response of patients might be explained by our findings from the standpoint of inflammatory complexes genes.

### 3.6 Increased expression of IFC genes could sensitize ccRCC patients to anti-tumor drugs

**Figure 6:**
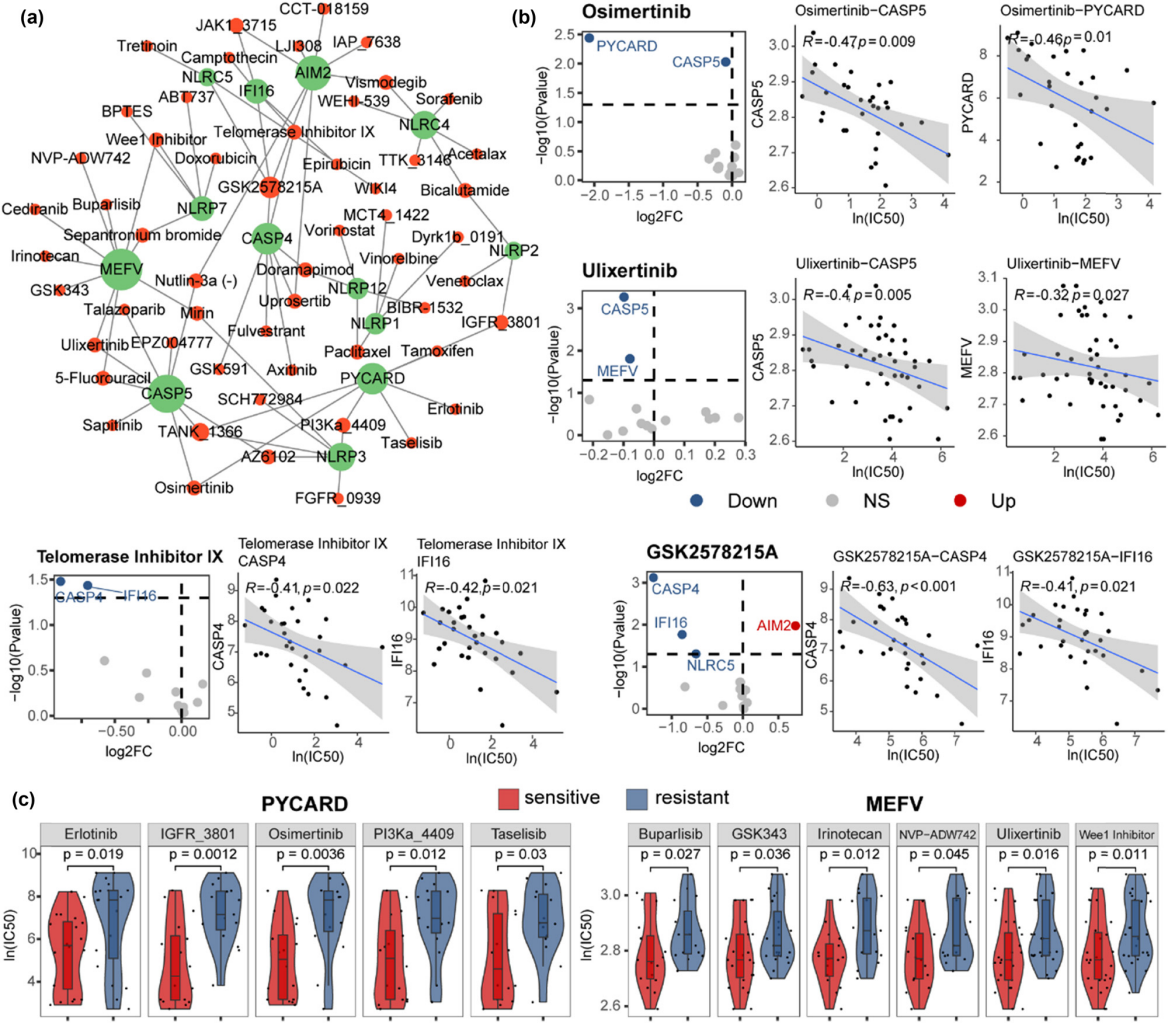
Increased expression of IFC genes could sensitize ccRCC patients to anti-tumor drugs. (a) Network shows the interactions between anti-tumor drugs and predicted target IFC genes. (b) Volcano plots show the expression variation of IFC genes between sensitive and resistant groups of each drug. Red nodes represent IFC genes that were up-regulated in drug-sensitive groups, while blue nodes represent down-regulated. Correlation curves depicting the relationship between the expression levels of IFC genes and drug sensitivity IC_50_ value in cancer cell lines. (Spearman’s correlation analysis, *p* < 0.05, |Rho| > 0.3). (c) Violin plots of gene expression between sensitive and resistant groups (Mann–Whitney *U* test *p* < 0.05).

Here, we aimed to predict potential drug targets for ccRCC treatment by IFC genes. Therefore, we explored the correlation between drug sensitivity information and the expression of IFC genes in ccRCC cell lines, constructing a network of drug–target interactions (Figure 6). Through this investigation, we found that the expression perturbation of IFC genes could lead to the resistance of several drugs which have been used for renal cell carcinoma treatment in clinical trials including targeted therapy drugs Axitinib [47,48,49,50], Sorafenib [51], Pazopanib [52,53], Sunitinib [54], and immunotherapy drugs Bevacizumab [55,56], Interleukin-2 [57,58], Pembrolizumab [47], Nivolumab [59,60]. Additionally, we identified several potential anti-tumor drugs for ccRCC treatment, including Osimertinib, Ulixertinib, Telomerase Inhibitor IX, and GSK2578215A (Figure 6a and b). Osimertinib is a chemotherapy drug which has been widely used for metastatic EGFR-mutant non-small-cell lung cancer treatment [61]. Ulixertinib is an ERK1/2 inhibitor that has been clinically tested effective for patients with advanced solid tumors [62,63]. Telomerase Inhibitor IX has the potential to inhibit the proliferation of cancer cells because of the abnormally high telomerase activity in cancer cells [64]. GSK2578215A is an *LRRK2* inhibitor that could affect the sensitivity of ovarian cancer treatment by suppressing homologous recombination [65]. Our results suggested the potential of these anti-tumor drugs in ccRCC treatment by targeting inflammasome signaling. Among IFC genes, we found that *MEFV* and *PYCARD* were predicted to be the targets of most drugs in the network. The increased expression level of these two IFC genes could sensitize ccRCC cells to various therapy methods (Figure 6c). In summary, our results indicated a potential role of the inflammasome pathway in modulating ccRCC patients’ response to these specific drugs.

## 4 Discussion

ccRCC is a multifaceted cancer with complicated biological and clinical features. As a key hallmark of cancer, inflammation has been shown to be closely related to the progression and prognosis of various tumors. In some cases, inflammation can promote the occurrence and development of tumors, which supports the therapeutic potential of targeting inflammation for cancer [66]. As the core of inflammation, the inflammasome, a multiprotein complex within cells, plays a vital role in initiating and regulating inflammatory responses, and the precise regulation of its function is significant for maintaining the body’s immune homeostasis [67]. The activation of inflammasome complexes is an important regulatory node in the inflammatory response. It can sense a variety of abnormal signals and regulate the intensity and duration of the inflammatory response through downstream signaling pathways [68]. In this study, we comprehensively investigated the expression patterns and clinical significance of inflammasome complexes in ccRCC, providing insight into their role in tumorigenesis, prognosis, and therapeutic response.

We found that most inflammasome complexes genes showed a significantly up-regulated pattern in ccRCC samples, which was further validated by immunohistochemistry (Figure 1b). Previous studies have established a positive correlation between high levels of inflammasome complexes and the development of cancer; the overactivation of inflammasome signaling in some cancer types is linked to the persistence of inflammatory responses, thereby promoting tumor formation and progression [10,11]. Our results further suggested the role of inflammasome complexes during the tumorigenesis of ccRCC. Moreover, our results showed that perturbations in inflammasome complex genes were associated with patient prognosis and other clinical characteristics by developing an IFC score and identifying IFC subtypes. Therefore, we provided evidence of inflammasome complexes genes’ clinical relevance in ccRCC and highlighted the potential of inflammasome complexes as prognostic markers for patient outcomes.

In addition, we found that there was a positive correlation between inflammasome signaling and immunosuppressive macrophages (Figure 5), indicating that inflammasome activation might contribute to an immunosuppressive tumor microenvironment, thereby facilitating tumor immune evasion and progression. These findings further validated the hypothesis that patients with lower IFC scores would be more likely to respond to immunotherapy (Figure 4f). Our study provided insights into the interplay between inflammasome complexes and the immune response in ccRCC. Significantly, our research also revealed the potential of inflammasome complex genes as predictive markers for patient responses to different anti-tumor drugs, such as GSK2578215A, Ulixertinib, Osimertinib, and Telomerase Inhibitor IX (Figure 6). This suggested that inflammasome activation may have implications for treatment selection and personalized medicine in ccRCC, which also implied the potential mechanisms by which inflammasome complexes modulate therapeutic response.

In summary, the results of our study demonstrated the clinical significance of inflammasome complexes genes in ccRCC and their potential as prognostic indicators and therapeutic targets. Our findings highlighted the importance of understanding the molecular mechanisms underlying inflammasome activation and its impact on tumorigenesis, the immune landscape, and treatment response in ccRCC.

## Supplementary Material

Supplementary material
