# Supplementary material for: Inflammasome complex genes with clinical relevance suggest potential as therapeutic targets for anti-tumor drugs in clear cell renal cell carcinoma
Source: Open Life Sci. 2024 Nov 12;19(1):20220980. doi: 10.1515/biol-2022-0980 (PMC11588011; doi:10.1515/biol-2022-0980)
Supplement: Supplementary material [file biol-2022-0980-sm.pdf]

Supplementary material

Table S1: Baseline characteristics of 30 cases of ccRCC

Characteristics	Cases (%)	Mean	Range
**Sex**			
Male	17 (56.67%)		
Female	13 (43.33%)		
**Age**		52.6	34-77
**Location**			
Left	18 (60.00%)		
Right	12 (40.00%)		
**Tumor size (cm)**			
≤7 cm	18 (60.00%)		
＞7 cm and <10 cm	9 (26.67%)		
≥10 cm	3 (10.00%)		
**WHO/ISUP classification**			
Grade 1	4 (13.33%)		
Grade 2	19 (63.33%)		
Grade 3–4	7 (23.33%)		

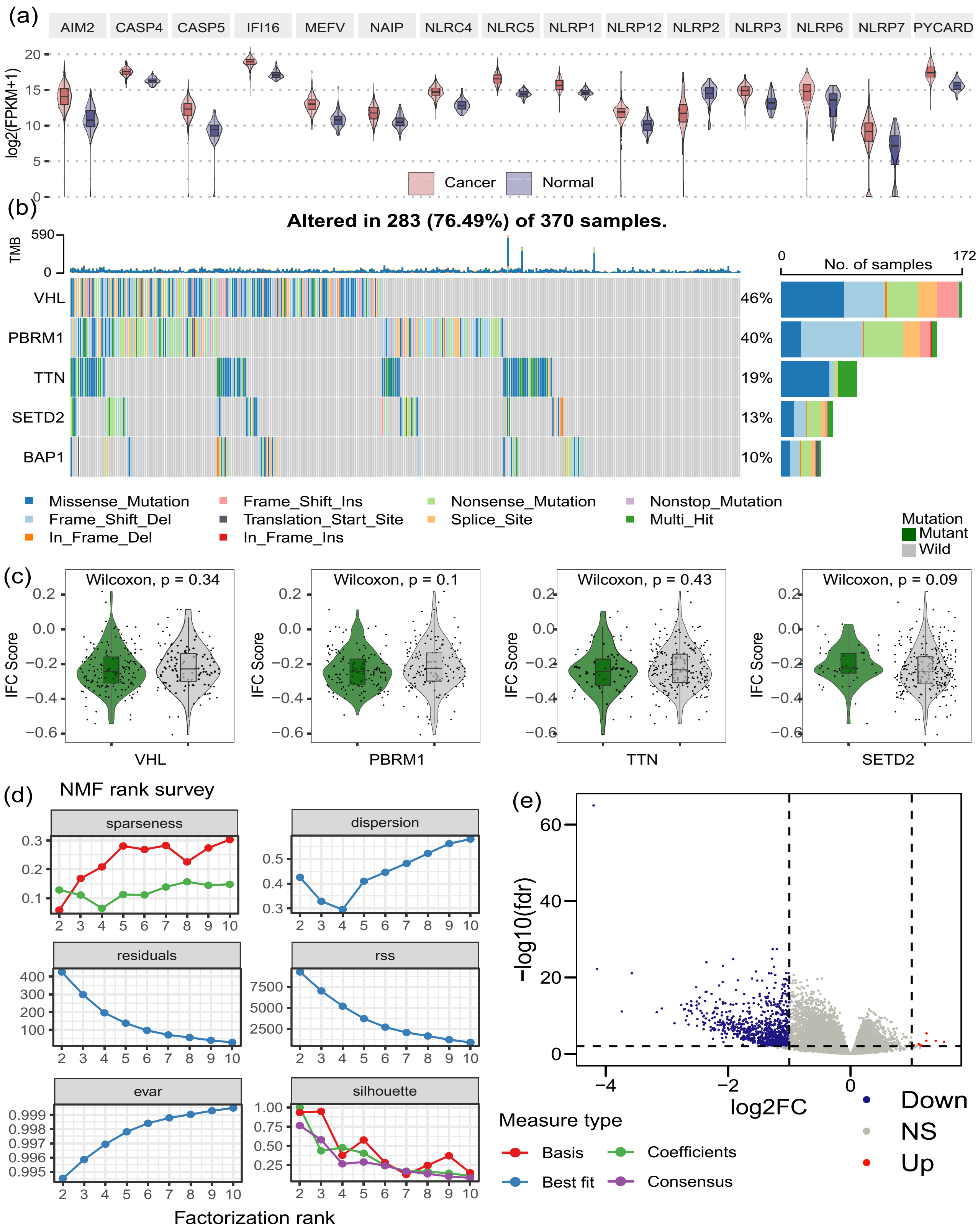

Figure S1: (a) Violin plots showing the expression differences of IFC genes between cancer and normal samples (Mann-Whitney U test $p < 0.05$). (b) Oncoplot of genes with high-frequency somatic mutation in ccRCC. (c) Violin plots shows the differences of IFC scores between mutant and wild-type samples. (d) Evaluation metrics of NMF performance ($k = 2{:}10$). (e) Volcano plot shows the dysregulated protein-coding genes between IFC subtypes.
